# Embedding public health intelligence into the global public health architecture

**DOI:** 10.1136/bmjph-2024-001011

**Published:** 2024-11-09

**Authors:** Neil J Saad Duque, Blanche Greene-Cramer, Adedoyin Awofisayo-Okuyelu, Dubravka Selenic Minet, Maria Almiron, Krista Swanson, Christian Hertlein, Thomas Mollet, Aura Corpuz, Etien Koua, George Sie Williams, Tamano Matsui, Manilay Phengxay, Masaya Kato, Tshewang Dorji, Silviu Ciobanu, Ka-Yeung Cheng, Oliver Morgan, Abdi Rahman Mahamud, Esther Hamblion

**Affiliations:** 1 Department of Alert and Response Coordination, Health Emergencies Programme, World Health Organization, Geneva, Switzerland; 2 Health Emergency Information & Risk Assessment, Health Emergencies, World Health Organization Regional Office for the Americas, Washington, District of Columbia, USA; 3 Health Emergencies Programme, World Health Organization Eastern Mediterranean Regional Office, Cairo, Egypt; 4 Health Emergencies Programme, World Health Organization Africa Regional Office, Brazzaville, Congo; 5 Health Emergencies Programme, World Health Organization Western Pacific Regional Office, Manilla, Philippines; 6 Health Emergencies Programme, World Health Organization South-East Asia Regional Office, New Delhi, India; 7 Health Emergencies Programme, World Health Organization European Regional Office, Copenhagen, Denmark; 8 WHO Hub for Pandemic and Epidemic Intelligence, Health Emergencies Programme, World Health Organization, Berlin, Germany

**Keywords:** Public Health, Epidemiology, Disease Outbreaks, Emergencies, Public Health Practice

 Acute public health threats occur continuously throughout the world and pose a risk to the health and well-being of people and populations. Early detection and rapid response to acute public health threats save lives, lessen the burden and limit health system or country-wide disruptions.^1^,^4^

One of the World Health Organization’s (WHO) key responsibilities is to prevent and respond to health emergencies,^5^ for which the organisation has developed a global public health intelligence (PHI) approach that includes detecting, verifying, risk assessing and reporting on acute public health threats. WHO’s PHI activities are underpinned by the International Health Regulations 2005 (IHR 2005), which came into force in 2007 (figure 1).^6 7^

**Figure 1:**
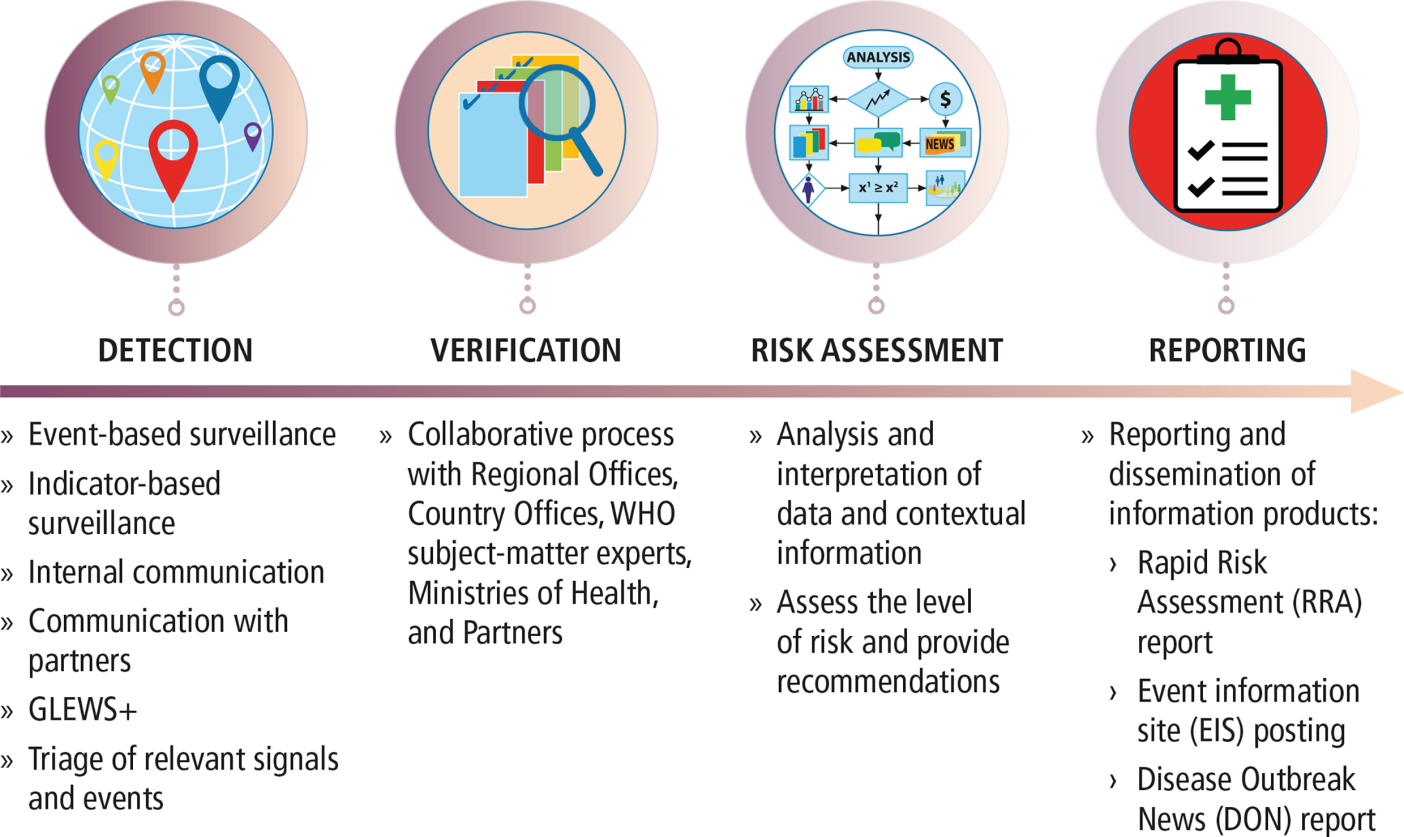
Framework of public health intelligence activities at the WHO. GLEWS+ is the Joint FAO–OIE–WHO Global Early Warning System for health threats and emerging risks at the human–animal–ecosystems interface.

PHI activities at WHO are conducted 24 hours per day, every day of the year, by dedicated teams at headquarters in Geneva and in the six WHO Regional Offices, in collaboration with WHO Country Offices, partners and national governments. In practice, the PHI teams continuously monitor multiple information channels to detect signals that could represent a potential acute risk to human health. Signals can, after verification with national governments, be designated as a substantiated acute public health event or simply an event. WHO prioritises those events that might be of international concern under the IHR (2005).^6 8^

The latest WHO global PHI report 2023 shows that in the last 20 years (2004–2023), 5910 events were recorded in WHO’s event management system (EMS), ranging from 159 to 463 events per year with a median of 280 events annually (figure 2). In 2023, 365 events were recorded, of which the majority (79%, n=289) were due to infectious diseases.^9^

**Figure 2:**
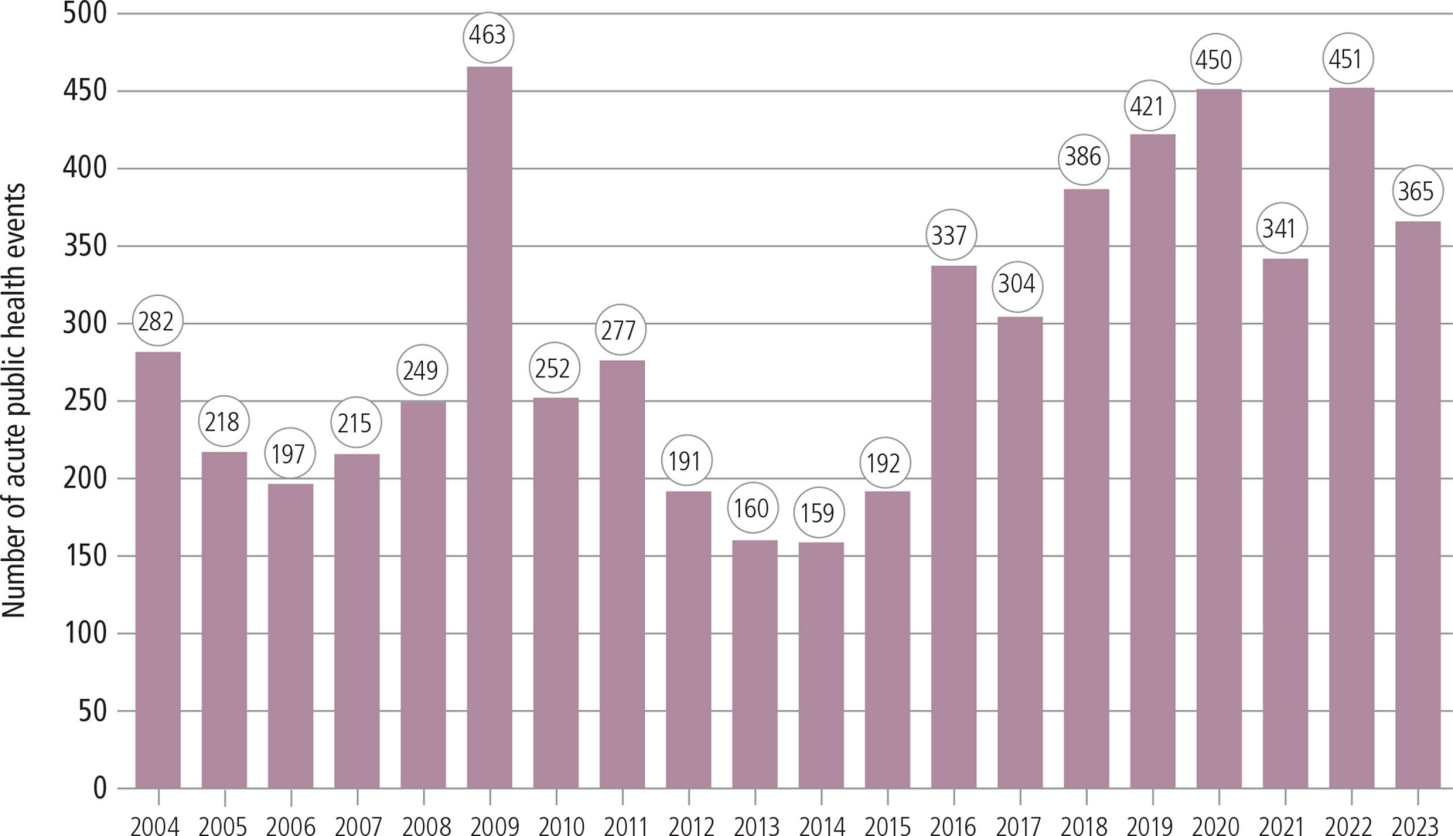
Acute public health events reported under the International Health Regulations (2005), 2004–2023.

Risk assessment is conducted for all events in EMS and reassessed when new information becomes available. Since 2017, a further in-depth risk assessment, the formalised WHO rapid risk assessment (RRA) is conducted for events that may require a WHO response.^10^ An RRA report informs decision-making and facilitates response actions by WHO, in collaboration with national governments and partners, including whether the event should be referred to an Emergency Committee for review as a public health emergency of international concern (PHEIC) . A total of 52 RRA reports were conducted globally in 2023, mostly related to dengue, cholera and measles.^9^

WHO alerts WHO Member States by sharing information on acute public health events through its Event Information Site for National IHR Focal Points (EIS). This platform has been developed by WHO to facilitate secure communications with the IHR National Focal Points as part of the implementation of the IHR (2005). In addition, information is also shared with the public through disease outbreak news (DON) reports and dedicated WHO web pages. This enables national governments and the public to receive authoritative and verified information in a timely fashion.^11^ In 2023, more than 125 EIS bulletins or announcements and 68 DON reports were published.^9^

The global PHI report 2023 underscores the importance of PHI in detecting, risk assessing and responding to acute public health events. WHO’s work in this area is facilitated by WHO’s regional offices, located in the Americas, Asia, Europe, Middle East and Africa and is complemented by numerous other public health actors who are also engaged in PHI activities at the regional and national level, including, for example, the European Centre for Disease Prevention and Control or the Africa Centres for Disease Control and Prevention.^12 13^

PHI’s speed of operation at WHO can be incredibly swift, in a matter of hours a team of PHI operators can move from detection to information dissemination. However, one of the rate limiting steps is the verification of signals. This is always conducted as rapidly as possible but might be delayed when a signal pertains to a more remote area or a (post-)conflict affected setting. Nonetheless, WHO also leverages verification through other actors and, in case of delays to verification, will always balance the need of sharing information timely while ensuring that it is robust and fact based.

WHO’s PHI system has both strengths and weaknesses (box 1) but the system is continuously being improved to enhance early detection and response. In addition, several ongoing trends will facilitate further improvements in PHI activities globally. First, the increasing abundance of data and information, including complementary information sources related to genomic, meteorological, environmental and migration data, will allow for more putative signals to be considered and provide more context and insight.^14^,^16^ Second, advances in automated analytics and artificial intelligence will enhance filtering and selection of key pieces of information to facilitate detection activities.^17^,^19^ Third, an upswell of networks by civil society, professional societies and non-governmental actors could facilitate verification of information detected, including in humanitarian settings where response activities can be hampered if a government presence is challenging.^20^ Finally, several ongoing endeavours to strengthen PHI capacity will contribute to the worldwide uptake of these activities.^21 22^ Having various public health actors involved in PHI activities produces a more effective global ecosystem for PHI which, in turn, provides different perspectives and redundancy for a system that needs to be highly reliable.

Box 1 **Strengths and weaknesses of WHO’s public health intelligence system**

Strengths:

- Continuous monitoring and verification of information globally.
- Robust risk assessment and rapid information dissemination to decision-makers, national governments and the public.
- Collaboration with a myriad of stakeholders, across national, regional, global and intergovernmental levels.

Weaknesses:

- Various surveillance systems and data in use globally limiting interoperability.
- Complexity of verifying information and information sharing in remote areas or (post-)conflict settings.
- Limited long-term and robust financing available.

Managing and sustaining PHI operations requires sufficient resources.^9^ At WHO, PHI operations require 12 types of distinct roles. These roles could be filled by multiple individuals per role or managed by combining roles for single individuals, depending on the scope of PHI activities.^6^ The overall cost of PHI operations, therefore, will depend on the scope and the local cost of skilled human resources and of infrastructure, such as IT equipment, office space and information management systems. However, in a resource-constrained situation for public health,^23 24^ greater systems efficiency can be achieved by having the different components of surveillance systems work together in a more collaborative way, which results in greater opportunities for effective PHI. Countries with limited financial resources should focus on strengthening public health surveillance, a key pillar of the detection component of PHI, and on furthering relations with global and regional public health actors engaged in PHI. These public health actors could leverage the multisource surveillance data from national governments for PHI activities and share the acquired insights to enhance timely decision-making at the national level. One example of information sharing is WHO’s publicly available global health emergency dashboard which provides quasi-real-time information on acute public health events and emergencies.^25^ In a global environment with dwindling resources, collaborative action will become ever more needed.^26^

A major impediment to wider PHI adoption and implementation is that PHI’s success is generally invisible, in contrast to failures or delayed action which are more easily discernable. It is harder for policy-makers and decision-makers to justify support for activities that are enumerated in cases averted, deaths not occurring, or as outbreaks that did not spread further after initial detection. Public health leaders must continuously reinforce the message that support for preventative action reduces disease burden, mitigates loss of life, limits disease spread and is an investment for health. By all accounts such rewards, including wider societal benefits, are invaluable. Actionable improvements for policy-makers and decision-makers should occur both within countries and in the context of multilateral and global collaborations. Key actions could include furthering automated analytics and artificial intelligence (including for enhanced contextual risk assessments), improving surveillance systems data interoperability, streamlining information sharing, leveraging complementary capabilities and investing in training and capacity building for PHI teams.

Current PHI approaches are developing rapidly due to advances in data science and more abundant data streams while a growing number of governmental, non-governmental and academic stakeholders working on PHI is creating a dynamic intelligence ecosystem. Sustained funding and support for PHI activities are essential in a world with a mounting number of public health threats, as is linkage to decision-makers. The latter is key to making PHI operationally successful and, therefore, WHO is increasing efforts to support WHO Member States and partners. Wider collaboration with and implementation of PHI activities is needed and is possible through building PHI capacity into existing national surveillance architectures using a collaborative surveillance approach across largely vertically managed disease control programmes.^26^ A strong and robust PHI infrastructure firmly embedded in the public health architecture everywhere will help tackle public health threats anywhere.

## Data Availability

Data sharing not applicable as no datasets generated and/or analysed for this study.
